# Advancements and Prospects of Metal-Organic Framework-Based Fluorescent Sensors

**DOI:** 10.3390/bios15110709

**Published:** 2025-10-24

**Authors:** Yuan Zhang, Chen Li, Meifeng Jiang, Yuan Liu, Zongbao Sun

**Affiliations:** 1 Department of Food & Biological Engineering, Jiangsu University, Zhenjiang 212013, China; 2 Food and Drug Supervision and Inspection Center in Zhenjiang, Zhenjiang 212000, China

**Keywords:** metal-organic frameworks, fluorescent sensor, nanozyme, luminescence, pollutant detection

## Abstract

Metal-organic frameworks (MOFs), a class of crystalline porous materials featuring a high specific surface area, tunable pore structures, and functional surfaces, exhibit remarkable potential in fluorescent sensing. This review systematically summarizes recent advances in the construction strategies, sensing mechanisms, and applications of MOF-based fluorescent sensors. It begins by highlighting the diverse degradation pathways that MOFs encounter in practical applications, including hydrolysis, acid/base attack, ligand displacement by coordinating anions, photodegradation, redox processes, and biofouling, followed by a detailed discussion of corresponding stabilization strategies. Subsequently, the review elaborates on the construction of sensors based on individual MOFs and their composites with metal nanomaterials, MOF-on-MOF heterostructures, covalent organic frameworks (COFs), quantum dots (QDs), and fluorescent dyes, emphasizing the synergistic effects of composite structures in enhancing sensor performance. Furthermore, key sensing mechanisms such as fluorescence quenching, fluorescence enhancement, Stokes shift, and multi-mechanism coupling are thoroughly examined, with examples provided of their application in detecting biological analytes, environmental pollutants, and food contaminants. Finally, future directions for MOF-based fluorescent sensors in food safety, environmental monitoring, and clinical diagnostics are outlined, pointing to the development of high-performance, low-cost MOFs; the integration of multi-technology platforms; and the construction of intelligent sensing systems as key to enabling their practical deployment and commercialization.

## 1. Introduction

MOFs are crystalline porous materials formed by the coordination of organic ligands with metal centers (metal ions or metal clusters) [1,2]. These materials possess a periodic network structure and combine the rigidity of inorganic materials with the flexibility of organic ones, often referred to as coordination polymers [3]. The metal ions (such as copper, zinc, iron, cobalt, aluminum, etc.) or metal clusters (such as Zn_4_O) act as nodes within the framework, dictating the chemical properties and structural stability of the MOF [4,5]. Organic ligands, typically organic molecules with multiple coordination sites (such as terephthalic acid, imidazole, pyridine, etc.), coordinate with metal ions to form two-dimensional or three-dimensional network structures [6,7]. The type of organic ligand and its mode of connection directly influence the pore structure, surface properties, and functional characteristics of the MOF [8,9]. This structural programmability extends to their photophysical properties, as the choice of metal centers (e.g., luminescent Ln^3+^ ions or transition metals involved in charge transfer) and organic ligands (e.g., conjugated molecules serving as antennas) fundamentally determines the origins of MOF fluorescence, which can stem from the ligand-centered, metal-centered, or charge-transfer states [10].

MOFs are composed of organic ligands as pillars and metal centers as nodes, allowing the classification of MOF materials into various categories, including IRMOFs [11,12], ZIFs [13,14], MILs [15,16], Porous Coordination Networks (PCNs) [17,18], and the University of Oslo (UiO) series [19,20,21,22]. Different types of MOFs can be interconverted by modifying their structure or altering one of their constituent elements [23]. Among these, IRMOFs are microporous crystalline materials formed by the self-assembly of discrete secondary structural units, such as the [Zn_4_O]^6+^ inorganic cluster, bridged by a series of aromatic carboxylate ligands in an octahedral arrangement [24]. ZIFs are MOFs with zeolite-like structures, synthesized by the reaction of Zn(II) or Co(II) with imidazole ligands [25,26,27]. MILs are obtained by combining various transition metals with dicarboxylate ligands such as fumaric acid and adipic acid. Coordination Pillared-Layer are constructed by coordinating hexacoordinate metal elements with neutral nitrogen-containing heterocyclic ligands, such as 2,2′-bipyridine, 4,4′-bipyridine, and phenols. The PCN series contains multiple cubic octahedral nanoporous cages, forming a cage-channel topological structure. UiO materials are characterized by zirconium-containing octahedral clusters [Zr_6_O_4_(OH)_4_], which are linked with twelve benzene-1,4-dicarboxylic acid (BDC) organic ligands, resulting in a three-dimensional microporous structure containing octahedral central cages and tetrahedral corner cages [28,29,30]. HKUST-1([Cu_3_(BTC)_2_(H_2_O)_3_]_n_), alternately referred to CuBTC or MOF-199. HKUST-1 is constructed from Cu^2+^ and 1,3,5-benzene tricarboxylic acid (H_3_BTC/BTC). With its paddle-wheel-like crystal lattice and many vacant metal centers, the compound has a highly porous, three-dimensional structure. This review mainly focuses on these six most common and frequently used MOF series for constructing fluorescence sensors based on MOFs. The common metal centers and ligands of these six MOF series are summarized in Table 1.

Due to the active metal sites, well-defined topological structures, high crystallinity, inherent porosity, and chemical stability of MOFs [31,32,33], these materials can mimic the catalytic centers and coordination environments of natural enzymes [34,35,36]. MOFs are often employed as nanomimetics in conjunction with biological enzymes for biosensing applications [37,38,39,40]. The tunability of MOF materials allows for the design and optimization of their performance by selecting different metal centers and organic ligands as needed [41,42]. This precise control is crucial for engineering fluorescence sensing mechanisms. By strategically designing ligands and selecting metal nodes, one can modulate electron transfer (e.g., Photoinduced Electron Transfer, PET) and energy migration (e.g., Fluorescence Resonance Energy Transfer, FRET) pathways within the MOF, which form the basis for the fluorescence response to analyses [43]. Compared to other traditional porous materials, MOFs exhibit an exceptionally high specific surface area and well-defined pore structures, enabling effective accommodation of guest molecules [2,44]. This unique feature underpins the versatility of MOFs and their broad range of potential applications.

Currently, high-performance liquid chromatography (HPLC) and gas chromatography (GC), combined with mass spectrometry (MS) or other advanced technologies, remain the preferred methods for detection across various fields. However, in underdeveloped laboratories or regions with limited resources, complex sample pretreatment, expensive laboratory equipment, and the need for highly trained personnel pose significant barriers to their implementation [45,46]. Therefore, there is an urgent need for detection strategies that are highly selective, accurate, and simple, with low costs [47,48,49]. Optical sensors based on molecular or nanostructured materials offer significant advantages, including short response times, ease of operation, low costs, and the potential for visual detection [29,50,51,52]. Notably, MOF-based fluorescent sensors have garnered increasing attention due to their high sensitivity to target analytes. Fluorescent emission from these sensors can arise from both the ligands and metal nodes, which can be finely tuned through interactions between the nanoparticles. Moreover, the incorporation of external materials into the MOF can induce changes in fluorescence intensity. Beyond intensity-based signals, the interaction with an analyte can alter fundamental photophysical parameters such as fluorescence lifetime and quantum yield. Monitoring these changes provides a robust sensing modality that can circumvent issues like probe concentration variations and background interference, offering enhanced reliability for quantitative analysis [53]. In summary, the composition of the MOF, the coordination environment of its metal nodes, its pore structure, and the interactions between the luminescent MOF and target molecules collectively contribute to a variety of fluorescence changes [54,55]. MOF-based fluorescence sensing technologies, due to their high sensitivity, rapid response, portability, and visual detection, have been widely applied in the detection of pathogenic bacteria [56,57,58], metal ions [59,60,61,62], pesticide residues [63,64,65,66,67], antibiotic residues [68,69,70,71,72], and toxins [73,74,75,76,77,78].

**Scheme 1:**
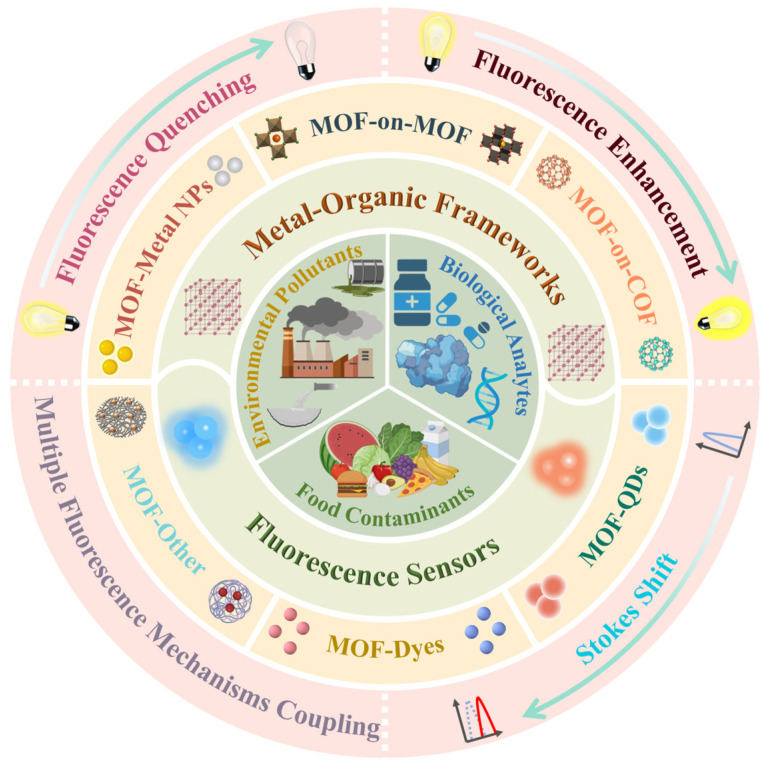
The application of fluorescent sensors based on metal-organic frameworks in rapid detection of biologically relevant analytes such as food contaminants, biological analyte, and environmental pollutants.

Despite their aforementioned remarkable advantages, the full exploitation of metal-organic frameworks (MOFs) in practical fluorescent sensing necessitates a systematic and in-depth understanding spanning material stability, diverse architectural design, and underlying photophysical mechanisms. This review aims to provide a comprehensive overview of the latest advancements in this rapidly evolving field. It will commence by systematically analyzing the degradation pathways that threaten MOF performance in practical applications and the corresponding stabilization strategies. Subsequently, the fluorescence sensing mechanisms of MOF-based sensors, including quenching, enhancement, Stokes shift, and multi-mechanism coupling, will be thoroughly elucidated with representative examples. Furthermore, the construction strategies of various MOF fluorescent sensor architectures—ranging from individual MOFs to sophisticated composites incorporating metal nanoparticles, other MOFs, COFs, QDs, and fluorescent dyes—will be detailed, highlighting the synergistic effects that enhance performance. The applications of these sensors in detecting biological analytes, environmental pollutants, and food contaminants will be extensively discussed to demonstrate their versatility and efficacy. Finally, the review will conclude by outlining future research directions and remaining challenges, pointing toward the development of high-performance, intelligent sensing systems for practical deployment in fields such as food safety, environmental monitoring, and clinical diagnostics (Scheme 1).

While previous reviews have, respectively, focused on specific photophysical mechanisms (e.g., Zhao et al. [79]), diverse functional roles in biosensing (e.g., Du et al. [80]), or the design of luminescent MOFs for targeted analytes (e.g., Dong et al. [81]), this review distinctively provides a systematic treatise that integratively links material stability, a broad spectrum of fluorescence mechanisms, and sophisticated multi-component sensor architectures.

## 2. Degradation Pathways and Stabilization Strategies for MOFs

MOF sensors face multiple severe chemical and physical challenges in practical applications, including water, acids/bases, anions, light, redox-active substances, and biofouling. These degradation pathways not only destroy their crystalline structure but also directly lead to the destabilization, drift, or quenching of fluorescence signals, severely limiting the sensors’ reliability, operational lifetime, and application scope. Therefore, an in-depth understanding of these degradation mechanisms is a prerequisite for “targeted treatment.” The following sections will first systematically elaborate on the main degradation pathways of MOFs, laying the groundwork for subsequently discussing targeted stabilization strategies.

### 2.1. Main Degradation Pathways of MOFs

#### 2.1.1. Hydrolysis

Liquid water or high-humidity environments represent the most prevalent threat to MOF sensors. Water molecules attack the metal/ligand coordination bonds, displacing the organic ligands through coordination substitution and leading to framework dissociation [82]. Taking the classic HKUST-1 (Cu-BTC) as an example, its coordinatively unsaturated Cu^2+^ sites possess high affinity for water molecules [83]. Water molecules attack the Cu-O bonds via a “push-and-twist” mechanism, ultimately causing the framework to collapse and transform into a non-porous hydrated phase [84]. For fluorescent sensors, framework hydrolysis not only leads to the failure of the pore structure but also directly causes fluorescence quenching or signal drift, resulting in complete sensor deactivation.

#### 2.1.2. Acid/Base Attack

MOF materials degrade under the influence of acids or bases, with mechanisms categorized as acid degradation and base degradation. Under acidic conditions, H^+^ competes with metal ions for coordination sites on the organic ligands, protonating the ligands (especially carboxylates) and breaking the metal/ligand bonds [82]. Under alkaline conditions, nucleophiles like OH^−^ readily attack and displace the organic linkers or bind to metal ions within the MOF clusters [85]. Studies have shown that MOFs composed of high-valent metal ions and carboxylate ligands exhibit significant stability in acidic environments but poor stability in alkaline environments, such as PCN-222 [86]. In contrast, MOFs composed of low-valent metal ions and nitrogen-containing azolate-based ligands are prone to decomposition under acidic conditions but demonstrate good stability in alkaline solutions, such as ZIF-8 [87]. In biosensing applications, pH fluctuations in bodily fluids require MOF sensors to possess a broad pH tolerance range; otherwise, their fluorescence signals will be interfered with by environmental pH [88].

#### 2.1.3. Ligand Displacement by Coordinating Anions

Strong coordinating anions in the environment (e.g., CO_3_^2−^, PO_4_^3−^, F^−^, EDTA) compete with the organic ligands in the framework for the metal nodes. These anions have a stronger binding affinity for the metal ions, thereby displacing the original ligands and destroying the framework structure. For instance, UiO-66 rapidly degrades in phosphate buffer solutions due to the high affinity between Zr and phosphate ions [89]; PCN-224(Co) decomposes in solutions containing F^−^ and CO_3_^2−^. Biological samples (e.g., cell culture media, serum) are rich in anions like phosphate, posing a severe challenge to the long-term stability of MOF sensors.

#### 2.1.4. Photodecarboxylation

Under prolonged sunlight (especially UV-Vis) irradiation, MOFs containing carboxylate ligands may undergo photocatalytic reactions, leading to ligand decarboxylation where CO_2_ is released. This causes framework defects and ligand loss. For example, UiO-66 and MIL-101(Fe) release CO_2_ after prolonged irradiation, although their crystal structures show no significant changes in XRD patterns, indicating a localized and gradual degradation process [90]. In contrast, MOFs without carboxylate ligands, such as the ZIF series, often exhibit excellent stability under prolonged sunlight exposure [91]. Photodegradation directly leads to the attenuation of sensor signals (photobleaching) and increased background noise, representing a key factor affecting their long-term photostability and service life.

#### 2.1.5. Redox-Driven Degradation

The mechanisms of redox-driven degradation can be primarily divided into reductive degradation and oxidative degradation. In anaerobic environments or in the presence of reducing agents, metal ions within the MOF are reduced, leading to changes in the coordination environment, weakened bond energy, and, ultimately, structural collapse. In applications such as advanced oxidation processes, generated reactive oxygen species (ROS) attack and oxidatively decompose the organic ligands, particularly those with electron-donating groups. When HKUST-1 was used to inhibit Saccharomyces cerevisiae, Cu(I) formed on its surface in the yeast culture, accelerating its degradation [92]. The tetrahedral Co sites in ZIF-67 are oxidized and transformed into CoOOH during electrocatalytic processes, completely destroying the structure [93]. The presence of various redox-active substances in biological systems (e.g., ascorbic acid, ROS) may trigger MOF degradation, leading to biocompatibility issues such as metal ion leakage and interfering with fluorescence detection signals.

#### 2.1.6. Biofouling and Biomolecule Adsorption

In complex biological media (e.g., serum, urine), biomacromolecules such as proteins and polysaccharides non-specifically adsorb onto the MOF surface or block its pores [94]. This biofouling not only hinders the diffusion of target analytes, reducing sensor response speed and sensitivity, but may also directly quench the fluorescence signal or generate non-specific background, leading to significant performance degradation or even sensor failure [95].

### 2.2. Stabilization Strategies for MOFs

#### 2.2.1. Constructing Strong Coordination Bonds

The core of this strategy is to fundamentally enhance the framework’s stability by selecting thermodynamically more stable metal/ligand pairs based on the Hard-Soft Acid-Base (HSAB) theory [96]. This involves pairing high-valent hard acid metals (Zr^4+^, Hf^4+^, Cr^3+^, Fe^3+^) with carboxylate hard base ligands, or low-valent soft acid metals (Zn^2+^, Ni^2+^) with nitrogen-containing azolate soft base ligands [97]. This is the cornerstone for constructing intrinsically stable MOF fluorescent sensors. For example, the 12-connected Zr_6_ oxo-cluster in UiO-66(Zr) forms extremely strong Zr-O bonds with terephthalic acid, making it exceptionally stable in water and strong acids, thus serving as an ideal platform for robust sensors [98]. The Zn-N bond formed between Zn and 2-methylimidazole in ZIF-8(Zn) possesses hydrophobicity and inertness, enabling long-term stability in boiling water and alkaline environments.

#### 2.2.2. Optimizing Framework Structure

The core of this strategy is to increase the kinetic energy barrier for framework dissociation, i.e., by optimizing the framework structure to significantly raise the activation energy for its dissociation reaction, thereby maintaining stability within the practical application timeframe [99].

First, increasing the connectivity of the nodes. The connectivity between metal nodes and organic ligands directly determines the framework’s robustness. Higher connectivity means more bonds need to be broken to remove one ligand, making the structure less prone to collapse [82]. The 12-connected Zr_6_ cluster in UiO-66 is the structural cornerstone of its exceptional stability [100]. Similarly, the 12-connected node composed of [Ni_8_(OH)_4_(H_2_O)_2_] units in PCN-601, together with tetratopic linkers, forms a high-density network, effectively suppressing the displacement of ligands by solvent molecules [101].

Second, employing short and rigid ligands. The rigidity of the ligand directly influences the energy of the dissociation transition state [85]. Short, rigid ligands require larger bond angle bending during dissociation, necessitating overcoming a higher energy barrier [101]. Research indicates that among porphyrin-based MOFs, PCN-601, which uses the shortest linker TPP^4−^, exhibits significantly higher kinetic stability than its counterparts using longer or more flexible linkers.

#### 2.2.3. Introducing Hydrophobicity and Protective Layers

This strategy does not alter the strength of the MOF’s chemical bonds but physically blocks destructive molecules like water and anions from contacting the active sites by introducing hydrophobic layers or protective coatings.

One method involves introducing hydrophobic functional groups (e.g., methyl, ethyl, trifluoromethyl) onto the MOF via post-synthetic modification or by directly using functionalized ligands. For example, the methyl groups in ZIF-8 impart hydrophobicity to its pores, increasing the relative pressure for water vapor condensation within the pores, thereby slowing the attack of water molecules on the [ZnN_4_] units [87]. After hydrophobic modification, MOF-303 can maintain structural integrity after immersion in various salt solutions (e.g., KCl, NaCl, MgCl_2_) for 50 days.

Another more general strategy is constructing a protective layer on the MOF surface. Covalent grafting of alkyl chains, fluoroalkyl chains, or polymer coating (e.g., polydimethylsiloxane, fluorinated polymers) can form a robust “protective shell.” Studies have shown that the water stability of HKUST-1 is improved by orders of magnitude after coating with a fluorinated polymer [83]. For fluorescent sensors, the protective layer can effectively shield fluorescence quenchers like water and oxygen, significantly enhancing the sensor’s photostability and service life. However, it is important to ensure that the introduced coating does not severely block the MOF pores, to avoid affecting analyte diffusion and sensing kinetics.

#### 2.2.4. Defect Control and Post-Synthetic Repair

Precisely controlling synthesis conditions (e.g., modulator ratio, temperature) or performing post-synthetic treatments (e.g., annealing at a certain temperature) can effectively reduce defects in the MOF, such as missing linkers or metal clusters [102]. Defects are initiation points for water molecules and chemical attacks; reducing defects can fundamentally enhance the intrinsic chemical stability of MOFs. For instance, UiO-66 subjected to post-synthetic annealing shows significantly enhanced water resistance and mechanical stability [103]. A complete, low-defect framework is an important guarantee for obtaining stable and reproducible fluorescence signals.

In summary, the stabilization strategies for MOF fluorescent sensors form a multi-dimensional, synergistic defense system against multiple degradation threats. Constructing strong coordination bonds serves as the cornerstone for enhancing the intrinsic stability of MOFs, fundamentally resisting hydrolysis, acid/base attack, and ligand displacement by coordinating anions. Optimizing the framework structure significantly increases the kinetic energy barrier for framework dissociation, effectively delaying processes such as hydrolysis, anion displacement, and redox-driven degradation. Introducing hydrophobicity and protective layers, as a physical barrier strategy, efficiently isolates the MOF from liquid water, water vapor, strong coordinating anions, and biomacromolecules, thereby avoiding issues like hydrolysis and biofouling, and indirectly improving photostability. Finally, defect control and post-synthetic repair eliminate weak points in the framework, blocking the initial attack sites for water, chemicals, and oxygen, providing a complete and solid structural foundation for all stabilization strategies. By comprehensively applying these strategies, high-performance MOF fluorescent sensors that maintain structural integrity and functional stability in complex chemical and biological environments can be systematically constructed.

## 3. Detection Mechanism of MOF-Based Fluorescent Sensors

MOF-based fluorescent sensors can detect target analytes by monitoring changes in the fluorescent intensity or wavelength of the luminescent MOF or other luminescent guest species. When the target molecule interacts with the active sites in the MOF sensor, it leads to alterations in the fluorescence signal. These changes can manifest as fluorescence quenching, fluorescence enhancement, or shifts in characteristic peaks, depending on the specific sensing mechanism and the nature of the target analyte [104].

### 3.1. Fluorescence Quenching

Fluorescence quenching occurs when certain chemicals or ions around the sensor molecules interact with the excited-state electrons, causing them to return directly to the ground state without emitting light [105,106,107]. This phenomenon is known as quenching. Quenching is commonly used to distinguish between similar substances or detect the presence of specific molecules [108,109]. For example, fluorescent quenching sensors are frequently employed for the detection of metal ions or gases [110].

Fan et al. developed a fluorescent MOF-0.02triethylamine (MOF-0.02TEA) sensor with a nanosheet microsphere morphology using coordination modulation to identify the three positional isomers of dihydroxybenzene. The fluorescence intensity of the MOF-0.02TEA sensor is quenched by the oxidation products of catechol and resorcinol, namely o-benzoquinone and p-benzoquinone, due to IFE, while remaining unaffected by hydroquinone. Furthermore, the introduction of o-phenylenediamine (OPD) further differentiates between catechol and resorcinol [111].

Yang et al. established a new method for determining Hg^2+^ and Cu^2+^ using a MOF/CdTe quantum dot sensor. The orange-red CdTe QDs gradually quench as the concentration of Hg^2+^ or Cu^2+^ increases, while the blue MOF remains unchanged, accompanied by a visual shift from pink to blue fluorescence [112].

He et al. developed a fluorescence sensor based on a Eu/Zr metal-organic framework (Eu_0.5_/Zr_0.5_-MOF) for the rapid detection of doxycycline (DOX) and L-arginine (Arg). Upon adding DOX, the fluorescence of Eu_0.5_/Zr_0.5_-MOF is quenched due to the IFE. However, the interaction between Arg and Eu_0.5_/Zr_0.5_-MOF@DOX weakens the IFE, restoring the fluorescence, thereby enabling the detection of Arg [113].

### 3.2. Fluorescence Enhancement

Certain substances can alter the structure of luminescent materials, thereby enhancing the emission intensity of fluorescence molecules. This phenomenon is known as fluorescence enhancement [114].

Deng et al. synthesized a dual-emission Eu metal-organic framework (Eu-MOF) using two organic ligands, 2-amino-terephthalic acid (BDC-NH_2_) and 1,10-phenanthroline, as a ratiometric fluorescence probe for the ultra-sensitive detection of aminoglycoside antibiotics (AGs). In this system, Eu^3+^ shows no sensitivity to AG concentration, and the red fluorescence produced serves as a reference signal, while the blue fluorescence of BDC-NH_2_ increases as the AG concentration rises. The strong hydrogen bonding between AGs, which contain abundant-NH_2_ and -OH groups, and the Eu-MOF facilitates the formation of intramolecular charge transfer in BDC-NH_2_, enhancing the blue fluorescence in the presence of AG solutions [115].

Miao et al. prepared a luminescent Tb-MOF as a signal label, which specifically binds to aptamers on the surface of Pt nanoparticles (Pt-Aptamer) to form a biosensor for non-invasive monitoring of Parkinson’s disease. The Pt-aptamer nanoparticle effectively quenches the luminescence of the Tb-MOF probe. Once the Pt-aptamer nanoparticles are removed, the probe exhibits a pronounced “on” fluorescence signal. Furthermore, as the concentration of Parkinson’s disease biomarker increases, the fluorescence of the sensor gradually enhances [116].

Yu et al. constructed a Mn-TCPP (BPDC) MOF for detecting moisture content. When the moisture content is low, water molecules exchange the BPDC ligand in the MOF, causing the 3D micron-sized biscuit-like structure to transform into 2D nanosheets and suppressing the ligand-to-metal charge transfer effect, which leads to fluorescence recovery. When the moisture content exceeds a certain threshold (based on different standards in organic solvents), the water molecules further exchange the porphyrin ligands, causing the MOF structure to dissociate and release the porphyrin, resulting in fluorescence enhancement [117].

### 3.3. Stokes Shift

When electrons in the excited state experience non-radiative energy loss, they return to the ground state. This process is accompanied by radiative transitions, where photons are released, resulting in fluorescence emission. Due to the energy loss, the wavelength of the fluorescence emission is always longer than that of the excitation light, typically manifested as a redshift.

Chen et al. reported a novel three-dimensional bimetallic MOF with strong fluorescence enhancement and a large redshift of 166 nm, which can serve as a rapid ratiometric fluorescence probe and a fast adsorbent for Pb^2+^ ions. In the presence of Pb^2+^, the fluorescence intensity of LMOF-1 significantly increased. The maximum emission peak of LMOF-1 shifted from 338 nm to 504 nm, resulting in a 166 nm redshift. When lanthanide metal ion solutions were added to the LMOF-1 suspension, only Tb^3+^ exhibited a unique emission peak, with the emission shifting from blue to green. Pb^2+^ exhibited a significant rapid “on” effect on LMOF-1, with the redshift in LMOF reaching a maximum of 166 nm, making it a promising candidate for use as a fast ratiometric fluorescence probe for Pb^2+^ detection [118].

### 3.4. Multiple Fluorescence Mechanisms Coupling

Target molecules can induce changes in the signals of multiple fluorophores, where the presence of the target substance causes distinct optical property variations in the responding fluorophores. For example, one fluorophore might experience enhanced fluorescence intensity due to specific interactions with the target (such as energy transfer, electron transfer, etc.), while another fluorophore could undergo fluorescence quenching due to similar reasons. This allows for the detection of a single target or the simultaneous monitoring of multiple targets, making it suitable for various complex real-world applications [119,120].

Lu et al. developed a smartphone-assisted dual-color ratiometric fluorescence smart gel tag visual sensing platform, where green-emitting CDs and blue-emitting bimetallic MOF (Fe/Zr-MOF) were coupled to form a dual-color CDs@Fe/Zr-MOF fluorescence nanoprobes as responsive signals. Experiments confirmed that Fe/Zr-MOF suppressed the fluorescence of CDs. As the concentrations of spermine and histamine increased, the internal filtering effect and electrostatic interactions of Fe/Zr-MOF caused a decrease in fluorescence intensity, with a slight redshift in the fluorescence peak of CDs due to an increase in the number of single-bond C and double-bond N groups. The suppressed fluorescence was recovered, resulting in fluorescence enhancement [121].

Kong et al. designed a novel dual-emission ratiometric fluorescence nanosystem based on green-emitting terbium MOF (Tb-MOF) and red-emitting bovine serum albumin-capped AuNCs (BSA@AuNCs) for the detection of heparin and chondroitin sulfate. With the addition of Hep and CS, the fluorescence of Tb-MOF was quenched, while the fluorescence of BSA@AuNCs continued to increase [122].

Chen et al. proposed an array-on-a-MOF fluorescence array, composed of a single MOF sensor array with different emission channels, for the sensing and identification of nitroaromatic compounds. This sensor integrates a lanthanide ion (Eu^3+^), a fluorescent dye (7-hydroxycoumarin-4-acetic acid, HCAA), and a luminescent UiO-66-type MOF. NACs primarily quench the fluorescence array via PET quenching of the UiO-66 (10) channel, energy competition at the Eu^3+^ channel, and IFE quenching at the HCAA channel [123].

Wang et al. designed a ratiometric fluorescence method for detecting dopamine and reduced glutathione. PDA-PEI can quench the fluorescence of UiO-66-NH_2_ MOF through FRET, while the fluorescence intensity at 530 nm gradually increases. However, when reduced glutathione is added, its reducing properties prevent dopamine and PEI from transforming PDA-PEI into oxidized glutathione, thereby restoring fluorescence at 450 nm and reducing fluorescence at 530 nm. This enables the detection of dopamine and reduced glutathione [124].

Xu et al. designed a ratiometric fluorescence biosensor based on acid phosphatase and hemin-loaded multifunctional zinc-based MOF (ACP/hemin@Zn-MOF) for high-performance arsenate (As (V)) sensing. The 2-amino-terephthalic acid ligand imparted inherent fluorescence to ACP/hemin@Zn-MOF (452 nm). During the oxidation of OPD catalyzed by ACP/hemin@Zn-MOF, a fluorescent 2,3-diaminophenazine (DAP) with an emission signal at 564 nm was produced. Due to the internal filtering effect, the inherent fluorescence of ACP/hemin@Zn-MOF (452 nm) decreased. After the addition of ascorbic acid 2-phosphate (AAP), ACP hydrolyzed AAP to generate ascorbic acid, which competitively inhibited the oxidation of OPD, leading to a decrease in DAP signal (564 nm) and a recovery of the ACP/hemin@Zn-MOF signal (452 nm). After adding As (V), ACP irreversibly inhibited the hydrolysis of AAP, resulting in the recovery of the fluorescence signal at 564 nm, while the signal at 452 nm was once again suppressed [125]. In recent years, examples of fluorescence sensors developed based on different types of MOF materials and their detection mechanisms are summarized in Table 2.

## 4. Strategies for Constructing MOF-Based Fluorescent Sensors

The combination of MOFs with various types of nanomaterials allows for the full exploitation of their respective advantages, thereby enhancing the performance of sensors, such as the amplification of fluorescent signals, improved selectivity, and increased stability. The choice of synthesis strategy plays a crucial role in determining the structure and properties of the MOF, which significantly impacts the overall performance of the sensor [152].

### 4.1. Individual MOFs

Sensing strategies based on individual MOFs rely on their intrinsic capability to integrate both recognition sites and signal units within a single framework. This approach typically utilizes the intrinsic luminescence of the MOF skeleton, which may originate from the emission of organic ligands, metal centers (particularly lanthanide ions such as Eu^3+^ or Tb^3+^), or sensitized emission mediated by the “antenna effect” between the two. Analytes diffuse into the pores or interact specifically with the framework surface (e.g., via coordination bonding, π–π stacking, or hydrogen bonding), thereby perturbing the frontier orbital energy levels, excited-state lifetime, or energy transfer pathways of the MOF. These interactions ultimately lead to either fluorescence enhancement (“turn-on”) or quenching (“turn-off”). The advantages of this strategy include structural homogeneity and straightforward synthesis. The underlying sensing mechanisms primarily involve PET, intramolecular energy transfer within the framework, and the inner filter effect, among others [72].

**Figure 1:**
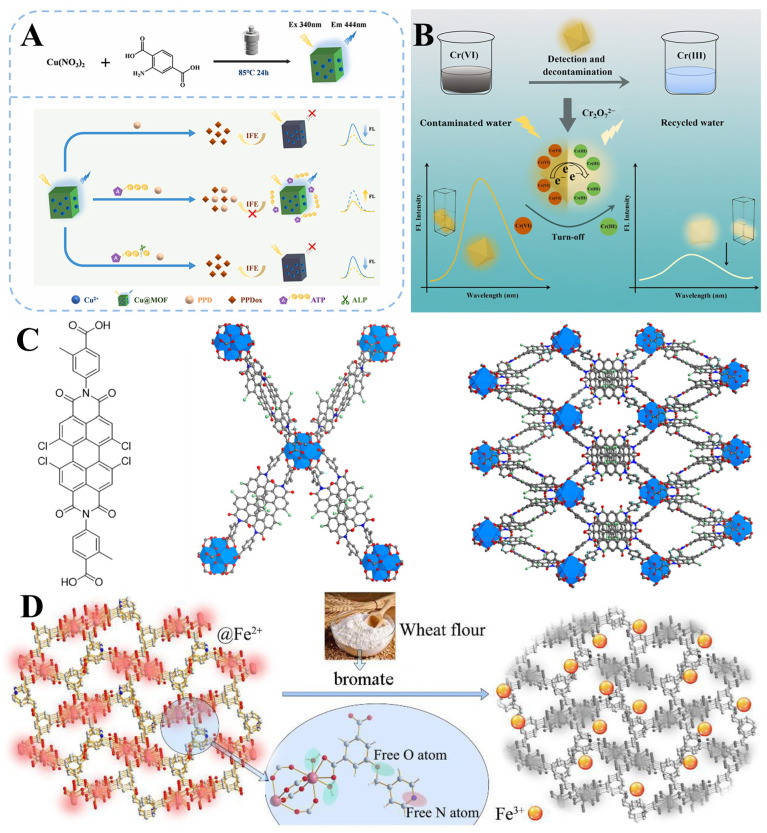
Examples of Individual MOFs for fluorescence sensing: (**A**) The illustration of the Cu@MOF synthesis and the mechanism diagram of fluorescence detection for ALP [153]. (**B**) Schematic diagram of Zr-MOF cleaning Cr (VI) from contaminated water and Zr-MOF synthesis and the mechanism of Cr (VI) detection and decontamination [154]. (**C**) Chemical structure of the PDI based ligand, H_2_L, used in this study, connection mode of Zr-cluster and molecular arrangement of the ligand, and a-Axis crystal structure of U-1 [155]. (**D**) The “turn off” Eu-MOF@Fe^2+^ sensor for fluorescence detection of bromate in wheat flour [156].

Chen et al. synthesized a bifunctional fluorescence sensor, Cu@MOF, using a hydrothermal method to detect alkaline phosphatase (ALP) with adenosine triphosphate (ATP) (Figure 1A). The sensing mechanism is rooted in the competitive coordination chemistry involving the Cu^2+^ sites within the MOF. Initially, the oxidase-mimetic activity of Cu@MOF catalyzes the oxidation of p-phenylenediamine (PPD) to PPDox, which quenches the intrinsic blue fluorescence of Cu@MOF via the IFE. The introduction of ATP restores the fluorescence because the phosphate groups of ATP strongly coordinate with the Cu^2+^ centers, thereby inhibiting the oxidase activity and preventing PPD oxidation. Conversely, when ALP is present, it hydrolyzes ATP into adenosine and inorganic phosphate, which disrupts the Cu^2+^-ATP complex. This liberation of Cu^2+^ reinstates the nanozyme’s catalytic activity, leading to the subsequent oxidation of PPD and the resultant fluorescence quenching. This reversible “on-off” fluorescence switching, governed by the presence of ATP and ALP, provides a highly sensitive and selective platform that has been successfully applied for ALP inhibitor screening and the determination of ALP activity in human serum samples [153].

Hu et al. synthesized a luminescent Zr-MOF using π-conjugated, electron-deficient, thiazole-based ligands with Zr (Figure 1B). The sensing mechanism for Cr(VI) (Cr_2_O_7_^2−^) is primarily attributed to the IFE, where the broad absorption band of Cr(VI) significantly overlaps with the excitation spectrum of the Zr-MOF, leading to competitive absorption of the excitation light and consequent fluorescence quenching. Additionally, the interaction between Cr(VI) anions and the functional groups on the Zr-MOF induces framework aggregation, further enhancing the quenching efficiency via an aggregation-caused quenching (ACQ) mechanism. Beyond its sensing capability, the Zr-MOF also serves as an efficient photocatalyst. Upon light irradiation, the organic ligand acts as an antenna to harvest photons, generating electron-hole pairs. The photogenerated electrons are then transferred to Cr(VI), facilitating its reduction to the less toxic Cr(III). This dual-functional Zr-MOF demonstrates great potential for both the detection and remediation of Cr(VI) pollution in water [154].

Rana Dalapati et al. designed and synthesized U-1 MOF, composed of a methyl-functionalized 1,2,6,7-tetrachloroperylene-3,4,9,10-tetracarboxylic acid diimide ligand (H_2_L) and the Zr_6_(μ_3_-O)_4_(μ_3_-OH)_4_ cluster (Figure 1C). The introduction of chlorine substitutions enhanced the solubility of the pentamethylene diisocyanate (PDI) ligand during the MOF synthesis process, while the incorporation of methyl groups increased the hydrophobicity around the Zr_6_ secondary building units within the MOF. This modification minimized interactions with water molecules while enhancing the MOF’s stability in aqueous environments. The detection mechanism of the U-1 MOF sensor is based on a fluorescence turn-on response triggered by its specific binding with the target molecule, perfluorooctanoic acid (PFOA). The core mechanism involves a synergistic effect: on one hand, the carboxylate group of PFOA strongly coordinates with the zirconium (Zr) metal clusters in the MOF; on the other hand, the perfluoroalkyl chain of PFOA engages in strong hydrophobic interactions with the chlorine-substituted hydrophobic backbone of the PDI ligand within the MOF. This dual-binding interaction significantly enhances the fluorescence intensity of the MOF, resulting in a transition from “weak fluorescence” to “strong fluorescence”. Consequently, the PDI-based MOF exhibits a rapid fluorescence turn-on response to PFOA in aqueous solution. This MOF sensor has been successfully applied for the detection of PFOA in tap water and drinking water. As a result, the PDI-based MOF rapidly activates a fluorescent response to PFOA in aqueous solutions. This MOF sensor has been successfully employed for the detection of PFOA in tap and drinking water [155]. Zhang et al. developed a luminescent europium-based metal–organic framework (Eu-MOF) via a facile one-step hydrothermal method (Figure 1D). The Eu^3+^ ions within this Eu-MOF exhibit strong red emission. Notably, its fluorescence can be efficiently quenched by Fe^3+^ but remains largely unaffected by either Fe^2+^ or bromate alone. Leveraging the strong oxidizing property of bromate (BrO_3_^−^), a turn-off sensor termed Eu-MOF@Fe^2+^ was constructed. In this system, BrO_3_^−^ oxidizes Fe^2+^ to Fe^3+^ through a redox reaction. The in situ generated Fe^3+^ subsequently quenches the fluorescence of the Eu-MOF. This quenching mechanism is attributed to the synergistic effect of coordination interactions between Fe^3+^ and the N/O-donor sites on the MOF ligand, along with competitive absorption of excitation energy. The sensor demonstrates high sensitivity and selectivity, and has been successfully applied for the detection of bromate in real wheat flour samples with satisfactory recovery rates [156].

### 4.2. MOF Composites

The advanced properties and applications of pure monomeric MOFs are limited due to their relatively simple composition, microstructure, and morphology [157]. Therefore, to enhance their functionality, MOFs are often combined with other materials to tailor their structural properties, stability in aqueous solutions, quenching efficiency, and fluorescence intensity, thereby enabling the fluorescence detection of target molecules. This approach involves either integrating MOFs with other functional units or assembling different MOFs into hybrid materials, thereby creating well-defined heterogeneous structures.

#### 4.2.1. MOF-Metal Nanomaterials

When metal nanomaterials (such as gold, silver, platinum, copper, etc.) are combined with MOFs [158,159,160,161], they can enhance the fluorescence signal through the local surface plasmon resonance effect, improving the sensitivity and selectivity of the sensors [162]. Metal nanoparticles not only amplify fluorescence but also enhance the sensor’s response to target molecules through interactions with the MOF surface [163,164].

**Figure 2:**
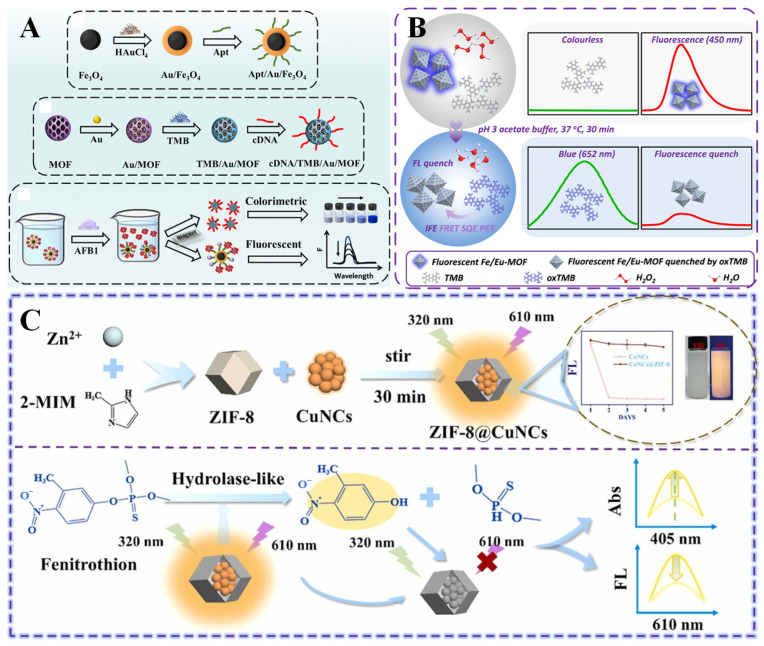
Examples of MOF-metal nanoparticle composite materials for fluorescence sensing: (**A**) Construction of the fluorescence/visual aptasensor and detection principle of the proposed dual-mode aptasensor [165]. (**B**) Schematic diagram of the influences of PPi and ALP on the fluorescence and peroxidase-like activity of Fe/Eu-MOF [166]. (**C**) Preparation of fluorescent nanozyme ZIF-8@CuNCs with hydrolase-like activity and the dual-mode detection of fenitrothion [167].

Liao et al. developed a fluorescence/visual aptamer sensor based on functionalized Au/MOF nanocomposites for the accurate and sensitive detection of Aflatoxin B_1_ (AFB_1_) (Figure 2A). The construction of this composite material aims to synergistically leverage the respective advantages of the MOF and metal nanoparticles: the MOF, with its exceptionally large specific surface area and ordered porous structure, serves as an efficient platform for loading the signal molecule TMB and the complementary DNA strand; meanwhile, the in situ reduced gold nanoparticles impart excellent peroxidase-like activity to the composite, which is crucial for catalyzing the subsequent colorimetric reaction. The detection principle is realized based on this material: initially, the recognition unit (Apt/Au/Fe_3_O_4_) and the signal probe (cDNA/TMB/Au/MOF) are assembled into a conjugate via hybridization between the aptamer and its complementary strand. Upon introduction of the target AFB1, it competes with the signal probe for binding sites on the aptamer, leading to the dissociation of some cDNA/TMB/Au/MOF probes from the conjugate into the supernatant. Following magnetic separation, the unconjugated complexes are used for fluorescence detection, where the fluorescence intensity decreases with increasing AFB1 concentration. Conversely, the released signal probes in the supernatant utilize the catalytic activity of their Au/MOF component to oxidize TMB in the presence of H_2_O_2_, producing a blue color, the intensity of which is proportional to the AFB1 concentration, thereby enabling visual quantification. Thus, the MOF/metal composite acts not only as the central hub for signal loading and amplification but also as the crucial bridge connecting the recognition event to the dual-mode fluorescence/visual signal output, thereby enabling highly sensitive, accurate, and convenient detection of AFB1 [165].

Shi et al. synthesized a dual-metal Fe/Eu-MOF with both fluorescent and peroxidase-like activity via a hydrothermal method (Figure 2B). The construction of this bimetallic MOF was designed to integrate the catalytic function of Fe and the fluorescence property of Eu within a single framework, and its sensing capability fundamentally relies on the strong affinity of pyrophosphate (PPi) for Eu^3+^ ions, which induces structural collapse and catalytic inhibition. Fe/Eu-MOF catalyzes the oxidation of the peroxidase substrate TMB to the blue oxTMB in the presence of H_2_O_2_. At the same time, the oxTMB produced in the solution quenches the fluorescence of Fe/Eu-MOF. Furthermore, the strong affinity between pyrophosphate (PPi) and Eu/Fe causes the dissociation of the Fe/Eu-MOF structure. Consequently, PPi significantly inhibits the peroxidase-like activity of Fe/Eu-MOF, leading to a decrease in absorbance and the restoration of fluorescence. As the effects of PPi and phosphate (Pi) on the catalytic activity of Fe/Eu-MOF differ, ALP can catalyze the hydrolysis of PPi to Pi, thus reducing the inhibition. This enables the construction of a dual-mode (colorimetric and fluorescence) ALP detection method based on MOF [166].

Li et al. constructed a composite fluorescent nanozyme, ZIF-8@CuNCs, using a zeolitic imidazolate framework-8 (ZIF-8) and copper nanoclusters (CuNCs) (Figure 2C). The construction strategy was based on the in situ encapsulation of fluorescent CuNCs within the pores of ZIF-8, which possesses organophosphorus hydrolase-like activity, achieving a stable composite via electrostatic interactions. This strategy not only significantly enhanced the fluorescence intensity and storage stability of the CuNCs but also endowed the composite with excellent hydrolase-mimetic activity. In detection applications, ZIF-8@CuNCs catalyze the hydrolysis of the target analyte, fenitrothion, producing a yellow product, 3-methyl-4-nitrophenol. This product exhibits a characteristic absorption at 405 nm, enabling colorimetric detection. Simultaneously, the hydrolysis product quenches the fluorescence emission of ZIF-8@CuNCs at 610 nm through an electron transfer mechanism, allowing for quantitative fluorescence detection. Based on this dual-signal output of absorbance and fluorescence, the sensor enabled highly selective and sensitive detection of fenitrothion, demonstrating potential for practical application in complex water environments [167].

The general design principles for MOF-metal composite sensing materials can be summarized as follows: through rational structural design, MOFs serve as ideal carriers that effectively immobilize and spatially confine metal nanoparticles, clusters, or ions by virtue of their high specific surface area, tunable pore size, and unsaturated metal sites. The metal components function as key functional units, providing catalytic activity, plasmonic effects, fluorescence emission, or acting as specific recognition sites to confer and enhance sensing capabilities. The synergistic interaction between the two components aims to efficiently convert the recognition event of the target analyte into a detectable signal output (e.g., optical or electrochemical signals). Through preconcentration effects, signal amplification, and stability enhancement, this strategy ultimately optimizes the sensor’s sensitivity, selectivity, and anti-interference ability.

#### 4.2.2. MOF-on-MOF

The MOF-on-MOF strategy constructs core–shell or heterojunction structures by integrating functionally distinct MOFs in an ordered manner at the nanoscale [168]. A general design principle of this approach is to leverage the differential fluorescence responses of different MOF components to the same analyte, thereby enabling the construction of self-calibrating ratiometric fluorescence sensors [169]. Typically, one MOF provides a stable reference signal, while the other supplies a response signal that varies with the analyte concentration [170]. This spatially separated functionalization not only prevents signal crosstalk but also facilitates synergistic signal modulation through internal energy transfer mechanisms—such as the IFE and FRET—thereby further enhancing the advantages of multiplexed detection and significantly improving the accuracy and reliability of the sensor [171].

**Figure 3:**
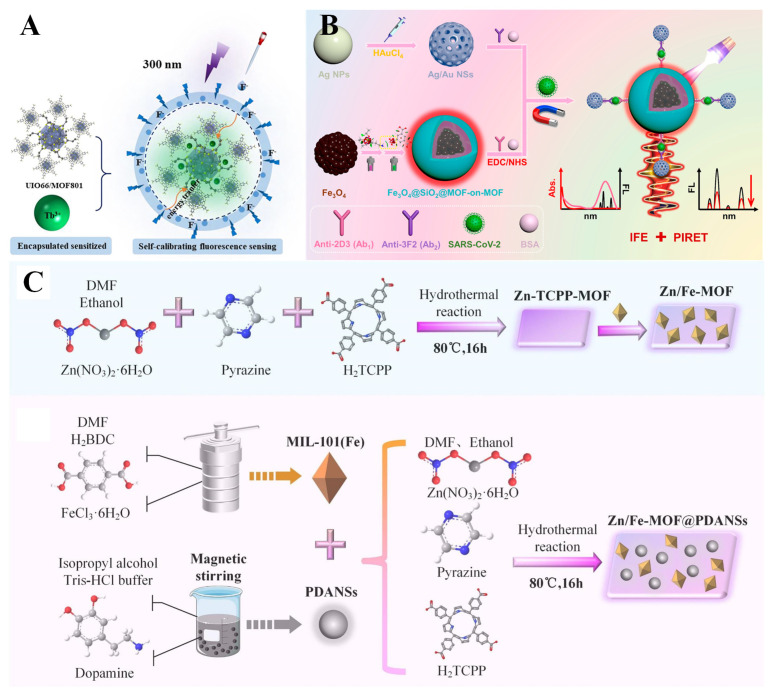
Examples of MOF-on-MOF composite materials for fluorescence sensing: (**A**) Schematic diagram of the UIO-66/MOF-801 encapsulated lanthanide ions with Tb^3+^ as an example [172]. (**B**) Schematic Illustration of Plasmonic DSA-Mediated Fluorescence Immunoassays Based on Magnetism-Functionalized Lanthanide MOF-on-MOF [173]. (**C**) Preparation of Zn/Fe-MOF@PDANSs [157].

Li et al. designed a fluorescence probe, Tb^3+^@UIO-66/MOF-801, by encapsulating sensitized Tb ions in UIO66-/MOF-801, which combined the strong fluorescence signal and water stability of both UIO-66 and MOF-801 (Figure 3A). The recognition mechanism for F^−^ is primarily attributed to the specific coordination interactions between F^−^ and the Tb^3+^/Zr^4+^ sites within the MOF. The introduction of F^−^ alters the coordination environment of Tb^3+^ and enhances the energy transfer efficiency from the organic ligands to Tb^3+^, thereby leading to a significant increase in the characteristic emission intensity at 544 nm. The emission peak at 544 nm originates from the Tb^3+^ ions, while the peak at 375 nm arises from the intrinsic luminescence of the MOF. In contrast, the intrinsic MOF emission at 375 nm remains unaffected by F^−^ and serves as an internal reference, enabling self-calibrating ratiometric detection of F^−^. The Tb^3+^@UIO-66/MOF-801 sensor exhibits high sensitivity towards F^−^, with a detection limit as low as 4.029 µM, which is much lower than the drinking water standard set by WHO. It also has a good linear response range and is suitable for precise detection of environmental water samples. This study highlights the tremendous potential of lanthanide-ion-encapsulated MOF-on-MOF materials as environmental sensors [172]. Hang et al. investigated the effect of different surfactants and active functional groups on the combination of magnetic nanoparticles with Ln-MOF to construct yolk-shell nanostructures through experimental results and theoretical calculations (Figure 3B). They confirmed that the strong intermolecular interactions between boronic acid-functionalized ligands and amino functional groups provided the optimal structure-activity relationship. Based on this, they designed a magnetic functionalized Fe_3_O_4_@SiO_2_@MOF-on-MOF system combined with plasma Ag/Au nanocages (Ag/Au NCs). The fluorescence enhancement effect of Fe_3_O_4_@SiO_2_@MOF-on-MOF and the efficient fluorescence quenching of Ag/Au NCs via FRET and IFE were used to detect the SARS-CoV-2 nucleocapsid protein (N protein). In the presence of SARS-CoV-2 N protein, antigen-labeled plasma Ag/Au NCs significantly reduced the fluorescence emission intensity of Ln-MOF. This study provides a new nanomaterial synthesis approach and signal amplification strategy for fluorescent immunoassays [173].

Wang et al. pioneered the development of a ratiometric fluorescence sensor array based on a metal nanocluster (MNCs)-immobilized MOF-on-MOF heterostructure (M@ZIF-on-MIL) for the efficient detection of multi-component perfluoroalkyl substances. In this study, a ZIF-8-on-NH_2_-MIL-101(AI) (ZIF-on-MIL) core–shell structure was synthesized via the epitaxial growth method, and gold nanoclusters (Au NCs) and gold-silver alloy nanoclusters (AgAu NCs) were successfully confined on its surface, significantly enhancing the fluorescence quantum yield of MNCs. The detection mechanism is distinctive: different PFAS molecules undergo metal coordination, hydrogen bonding, and hydrophobic interactions with the ZIF-8 shell, inducing different degrees of dissociation of the ZIF-8 structure. On one hand, this process releases Zn^2+^, which coordinates with the core NH_2_-MIL-101(AI), leading to fluorescence enhancement (“Turn-On”); on the other hand, the destruction of the ZIF-8 structure eliminates its confinement effect on the surface MNCs, resulting in fluorescence quenching of MNCs (“Turn-Off”). These simultaneous and opposite fluorescence changes generate a unique ratiometric fluorescence signal “fingerprint”. By combining a machine learning algorithm to recognize multi-dimensional signal patterns, the sensor array achieved 100% accurate discrimination of eight types of PFASs and could precisely detect PFAS samples with different concentrations and mixing ratios, also demonstrating excellent performance in complex environmental water samples. This work provides a novel paradigm for constructing multifunctional, self-calibrating MOF-on-MOF sensing platforms [174].

Liang et al. designed and synthesized a ternary MOF composite, Zn-TCPP-MOF@PDANSs@MIL-101(Fe) (Figure 3C), using water-soluble MIL-101(Fe), the active-site-rich Zn-TCPP-MOF, and the strong spectral overlap and quenching ability of polydopamine (PDANSs). They introduced 6-carboxyfluorescein (FAM)-modified ssDNA into the sensor for selective detection of sulfamethoxazole (SMZ). The prepared sensor exhibited dual fluorescence signals from Zn-TCPP-MOF and FAM-ssDNA. The ssDNA adsorbed onto the MOF, causing fluorescence quenching of FAM. When SMZ was present, FAM-ssDNA preferentially bound to SMZ and changed its conformation, blocking the FAM fluorescence quenching process, while the fluorescence of Zn-TCPP-MOF remained unchanged. In summary, this method calculates the concentration of SMZ by detecting the fluorescence signals of FAM and Zn-TCPP-MOF [157].

#### 4.2.3. MOF-COF

In most reported applications of MOFs, their limited fluorescence emission often fails to meet detection requirements, which hinders their use in biosensing applications. Hybrid materials combining MOFs and COFs can integrate the unique properties of both MOF and COF components [175]. COFs are materials with highly ordered structures, and by covalently linking MOFs and COFs into a core–shell configuration, new core–shell fluorescent materials (MOF@COF) can be obtained [176,177,178]. Nano-sized MOF@COF structures exhibit high porosity and large surface areas, providing more imprint sites and enhancing mass transfer rates [179].

**Figure 4:**
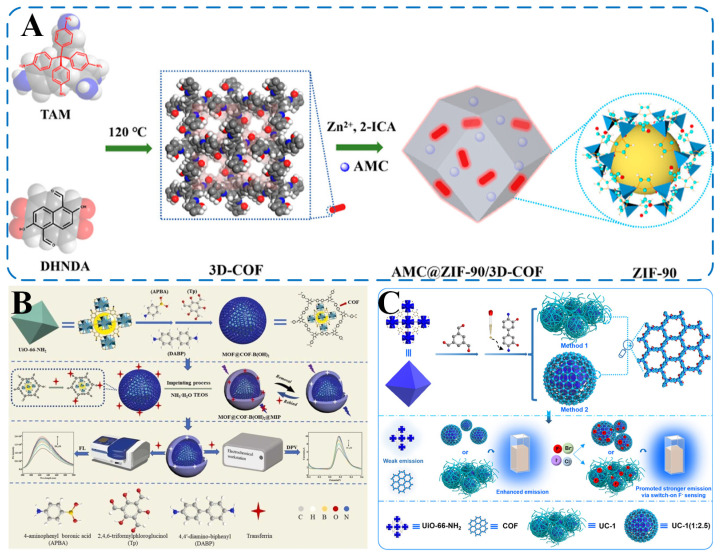
Examples of MOF-COF composite materials for fluorescence sensing: (**A**) Schematic diagram for synthesis of AMC@ZIF-90/3D-COF [180]. (**B**) Preparation of MOF@COF-B(OH)_2_@MIP and application in fluorescence-electrochemistry dual-mode analysis [181]. (**C**) Schematic preparation of COF-on-MOF UC materials and their luminescence performance [136].

A study grew ZIF-90 in situ on a porous 3D-COF to obtain a novel luminescent sensor, with 7-amino-4-methylcoumarin (AMC) encapsulated in the ZIF-90 framework (Figure 4A). The purpose of constructing this AMC@ZIF-90/3D-COF composite was to leverage the synergistic properties of its components: the MOF (ZIF-90) serves as a porous host for encapsulating and protecting the AMC dye, while the COF provides a stable, inherently fluorescent 3D scaffold. The AMC acts as the responsive fluorescent reporter unit. The fabricated AMC@ZIF-90/3D-COF could detect Chloroquine phosphate (CQP) in the “on” mode and folic acid (FA) in the “off” mode. At an excitation wavelength of 365 nm, in the presence of CQP, the emission intensity of AMC at 430 nm increased, while the emission intensity of 3D-COF at 598 nm remained almost unchanged. For FA detection, the fluorescence signal at 598 nm (from the 3D-COF) was selectively quenched, a process attributed to the inner filter effect and hydrogen bonding interactions between FA and the composite. This fluorescence quenching response showed high resistance to interference and selectivity for FA detection. This work presents a dual-functional optical platform for luminescent sensing and anti-counterfeiting applications [180].

Hu et al. designed and synthesized a boronic acid-functionalized porous framework composite (MOF@COF-B(OH)_2_), the UiO-66-NH_2_ core provides structural stability and intrinsic fluorescence, while the COF–B(OH)_2_ shell introduces abundant boronic acid groups, achieving specific recognition of specific glycoproteins through borate ester affinity interactions, thus eliminating the need for complex subsequent modifications. Using a surface-based molecular imprinting strategy, a dual-mode imprinted sensor (MOF@COF–B(OH)_2_@MIP) was fabricated (Figure 4B). For detection, the fluorescence mode operated based on photo-induced electron transfer, where binding of the glycoprotein transferrin (TrF) to the imprinted sites resulted in significant fluorescence quenching at 430 nm. In parallel, an electrochemical mode was established by modifying a screen-printed carbon electrode with MOF@COF–B(OH)_2_@MIP; the specific binding of TrF hindered electron transfer of the redox probe, leading to a measurable decrease in differential pulse voltammetry current. Due to its inherent fluorescence characteristics, the fluorescence intensity of MOF@COF–B(OH)_2_@MIP showed a good linear relationship with different concentrations of glycoproteins [181]. Based on the study by Xia et al., a core–shell MOF@COF composite was constructed by in situ growing a COF layer, synthesized from 1,3,5-benzenetricarboxaldehyde (BT) and 3,3′-dihydroxybenzidine (DH), onto the surface of UiO-66-NH_2_ MOF cores (Figure 4C). In this hybrid structure, the UiO-66-NH_2_ core served as a structural scaffold and provided intrinsic fluorescence, while the COF shell contributed extended π-conjugation. The restricted growth of the COF layer effectively suppressed ACQ, leading to significantly enhanced luminescence, with the optimal composite (UC-1) exhibiting a 7-fold increase in quantum yield compared to the bulk COF. The composite was further applied as a turn-on fluorescent sensor for fluoride ions (F^−^), where the detection mechanism was based on the inhibition of the excited-state intramolecular proton transfer (ESIPT) process in the COF layer upon interaction with F^−^, resulting in a progressive enhancement of fluorescence emission [136].

In summary, the synthesis of MOF@COF composites for sensing predominantly follows a core–shell strategy, where a COF layer is grown in situ on a MOF core. A key design principle is the functional synergy between the components: the MOF often provides a structural scaffold and intrinsic porosity, while the COF shell contributes specific recognition sites and, critically, its restricted growth mitigates aggregation-caused quenching to enhance luminescence. Furthermore, a recurring and powerful approach is the integration of multiple transduction mechanisms—such as ratiometric fluorescence and electrochemistry—within a single composite. This multi-modal design significantly improves detection reliability and broadens the application scope of these hybrid sensors, effectively addressing the limitation of weak or single signals from individual components.

#### 4.2.4. MOF-QDs

QDs are a type of nanomaterial with unique fluorescence properties, offering advantages such as high brightness, high stability, and tunable spectral features [182,183,184,185,186,187]. The combination of QDs and MOFs enhances the fluorescence signal of sensors through the optical properties of QDs [188], while the high surface area and functionalized surface of MOFs improve detection efficiency [189,190,191,192].

**Figure 5:**
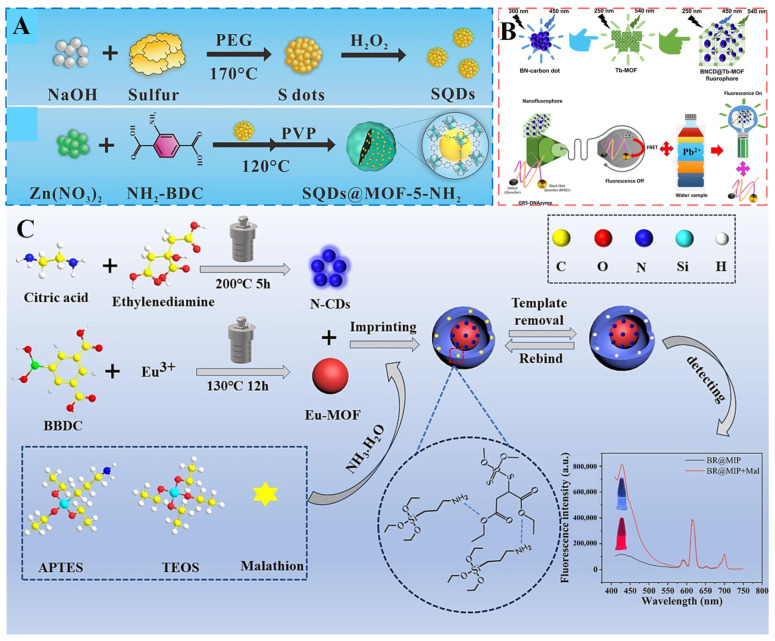
Examples of MOF-QDs composite materials for fluorescence sensing: (**A**) Illustration of the SQDs synthetic process and illustration of the SQDs@MOF-5-NH_2_ synthetic process [74]. (**B**) Schematic illustration of synthetic process of BNCDs@Tb-MOF [62]. (**C**) Schematic illustration of synthetic process of BR@MIP [193].

Yan et al. constructed a simple and effective self-circulating catalytic hairpin assembly (scCHA) and fluorescence aptamer sensor (SQDs@MOF-5-NH_2_), which encapsulated Sulfur quantum dots (SQDs) in MOF-5-NH_2_ (Figure 5A). SQDs were synthesized via a one-step hydrothermal method, then encapsulated in MOF-5-NH_2_ using a solvothermal process to form the SQDs@MOF-5-NH_2_ fluorescence probe. The system was further coupled with Fe_3_O_4_-NH_2_ and scCHA strategies to build a complete fluorescence aptamer sensing system, achieving cyclic magnetic capture of the luminescent signal in the presence of patulin (PAT). This system provides a dual-amplification strategy for the sensitive detection of PAT in apple juice. In this sensor, SQDs serve as the core fluorophore, while MOF-5-NH_2_ acts as their carrier, significantly enhancing the fluorescence intensity and providing sites for DNA immobilization. When the target PAT is present, it triggers the scCHA reaction, resulting in the magnetic capture of numerous SQDs@MOF-5-NH_2_ fluorescent probes by Fe_3_O_4_-NH_2_. By detecting the captured fluorescence signal, ultrasensitive detection of PAT is achieved [74]. Jain et al. combined a lanthanide-based metal-organic framework (MOF) with boron-nitrogen-doped carbon dots (BNCDs) to create a dual-emissive fluorescent signal transducer (Figure 5B). A lead-specific GR-5 DNAzyme system was employed as the recognition element. In this system, the BNCDs/Tb-MOF nanocomposite was covalently linked to the substrate strand of the DNAzyme, while the enzyme strand was labeled with a quencher (BHQ1). In the absence of the target, the close proximity between the fluorophore and the quencher, due to the hybridization of the two strands, quenched the fluorescence via the FRET process. Upon the addition of Pb^2+^, the DNAzyme was activated and cleaved the substrate strand. This cleavage released the fluorescent BNCDs/Tb-MOF fragment, leading to a significant recovery of fluorescence due to the spatial separation from the quencher. This mechanism enabled the ultrasensitive detection of Pb^2+^ in aqueous solutions [62].

Yang et al. developed a ratio-imprinted fluorescence sensor, N-CDs@Eu-MOF@MIP (BR@MIP), for the sensitive detection of malathion (Mal), using europium-based MOFs (Eu-MOF) as carriers to enhance the sensitivity of the BR@MIP sensor (Figure 5C). Nitrogen-doped carbon dots (N-CDs) were used as the fluorescence source to generate fluorescence signals. The new ratio fluorescence imprinted sensor (BR@MIP) based on Eu-MOF and N-CDs was constructed for the recognition of Mal. The red-emitting Eu-MOF and blue-emitting QDs (N-CDs) were used as reference and response signals, respectively. Eu-MOF served as the support and reference signal, improving mass transfer and sensitivity, while the MIPs on the Eu-MOF surface acted as the recognition sites for Mal. Mal. can bind to the recognition sites in the composite material, thereby specifically restoring the blue fluorescence that was quenched by the N-CDs. Due to the combination of Eu-MOF, N-CDs, and MIPs, the BR@MIP fluorescence sensor exhibited excellent visual response, sensitivity, and selectivity for Mal [193].

The construction of COF-QD composites for sensing primarily leverages a synergistic design. The robust and porous crystalline structure of COFs acts as a protective scaffold to host QDs, mitigating aggregation and enhancing stability. Simultaneously, the COF can be pre- or post-functionalized with specific recognition sites, which work in concert with the fluorescent QDs to create a highly selective and sensitive “signal-on” or “signal-off” sensing platform. This strategic integration combines the superior optical properties of QDs with the high surface area and molecular precision of COFs for effective detection.

#### 4.2.5. MOF-Dye

Fluorescent dyes are a class of chemical substances that can absorb light of specific wavelengths and emit light at different wavelengths [194]. They are widely used in fields such as biomedicine, environmental monitoring, and chemical analysis. Common fluorescent dyes include rhodamine, fluorescein, BODIPY, and aniline-based dyes [195,196,197]. These dyes exhibit strong fluorescence signals; however, their fluorescence performance is often influenced by the solution environment (e.g., pH, ion concentration, solvent effects), leading to unstable signals [198]. By encapsulating the fluorescent dye in the pore structure of MOFs, the MOF provides a protective environment that prevents the dye from decomposing or deactivating in external conditions [199]. Additionally, the structure of MOFs helps to disperse and stabilize the dye, ensuring that the fluorescence signal is generated stably over time [200,201].

**Figure 6:**
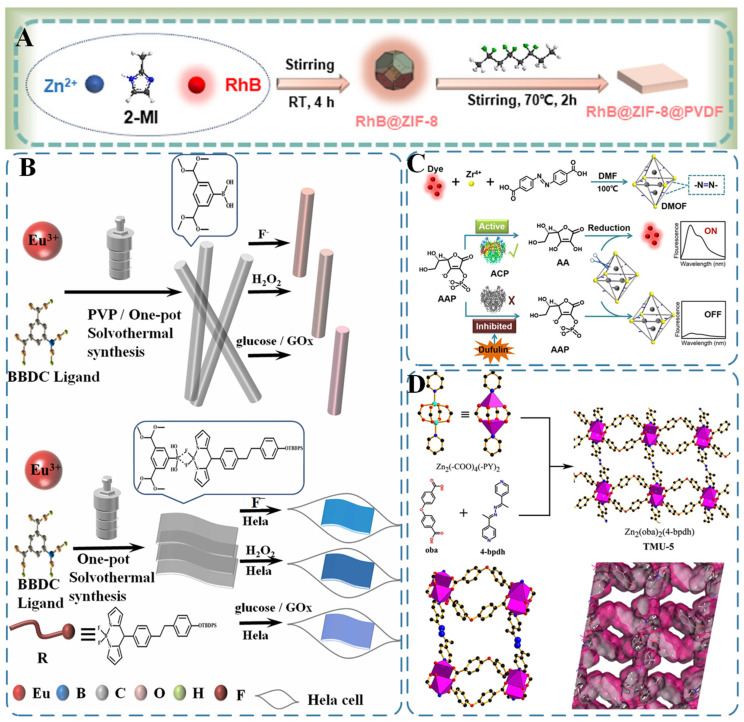
Examples of MOF-dye composite materials for fluorescence sensing: (**A**) Process for the preparation of RhB@ZIF-8@PVDF [202]. (**B**) Schematic illustration of the fabrication and the corresponding color change with ions of Eu-MOF and 5R@Eu-MOF sensor [203]. (**C**) Structure of TMU-5 (top) and the azine functionalized pores and 3D Connolly surface representation (bottom) [204]. (**D**) Construction of DMOF and the fluorescent sensing applications for acid phosphatase activity and its inhibitor Dufulin [205].

Xu et al. encapsulated the dye molecule rhodamine B (RhB) in the highly stable zeolitic imidazolate framework (ZIF-8) to obtain RhB@ZIF-8 composite luminescent materials. Polyvinylidene fluoride (PVDF) was chosen as the base polymer, and a phase conversion method was used to prepare the RhB@ZIF-8@PVDF composite membrane (Figure 6A). In the sensor, ZIF-8 prevents the aggregation of dye molecules, and the polymer limits the aggregation of the luminescent hybrid material, maintaining its emission characteristics. Therefore, RhB@ZIF-8@PVDF mixed-membrane matrix (RhB@ZIF-8@PVDF-MMM) has the luminescent properties of both the dye and the ZIF-8 ligand and can be used for ratio sensing. The sensor enables rapid detection of nitrofurantoin (NFT) and oxytetracycline (OTC). For NFT, its detection is based on a fluorescence quenching mechanism. The porous ZIF-8 adsorbs and enriches NFT molecules. Since the absorption spectrum of NFT highly overlaps with the emission spectrum of ZIF-8 at 436 nm, it selectively quenches the blue fluorescence of ZIF-8 through FRET and the inner filter effect, while the emission peak of the encapsulated RhB at 576 nm remains relatively stable. Whereas the detection of OTC is based on its coordination with Zn^2+^ in ZIF-8 to form a metal complex, resulting in fluorescence enhancement [202]. Li et al. develops a multifunctional ratiometric fluorescent sensor. The construction strategy involves grafting highly emissive BODIPY dyes onto a Europium-based MOF via covalent F-B bonding using a facile one-pot solvothermal reaction, forming the BODIPY@Eu-MOF composite. (Figure 6B) The detection principles for different analytes are as follows: For fluoride ions, sensing relies on the high affinity between F^−^ and the boronic acid group on BODIPY. This interaction alters the ligand-to-Eu^3+^ energy transfer efficiency, quenching the red emission of Eu^3+^ at 616 nm while enhancing the blue emission of BODIPY at 420 nm, enabling ratiometric detection. For hydrogen peroxide, its unique nucleophilic reaction with the boronic acid group cleaves the B-C bond, similarly perturbing the energy transfer pathway and causing a ratiometric fluorescence response. Glucose detection is achieved indirectly with high sensitivity by employing glucose oxidase to convert glucose into H_2_O_2_, which is then detected through the aforementioned H_2_O_2_ sensing mechanism [203].

Liu et al. developed a fluorescent nanosensor for detecting the pesticide Dufulin (Figure 6C). The construction strategy involves a one-pot synthesis to encapsulate the dye molecule (Doxorubicin, DOX) within a zirconium-based azobenzene-dicarboxylate Metal-organic Framework, forming the DMOF composite. The detection principle is based on a cascade reaction: The enzyme acid phosphatase (ACP) catalyzes the hydrolysis of its substrate AAP into ascorbic acid (AA). AA then reduces and cleaves the azobenzene units in DMOF, causing the framework to collapse and release the encapsulated DOX, thereby recovering fluorescence. In the presence of Dufulin, the activity of ACP is inhibited, blocking this reaction cascade and preventing fluorescence recovery. Consequently, the change in fluorescence signal allows for the highly sensitive detection of Dufulin [204]. Minoo Bagheri et al. introduced rhodamine B into the framework of TMU-5, synthesizing a two-dimensional fluorescent MOF sensor, TMU-5S (Figure 6D). The basic azine groups of the MOF enhance the quantum yield by interacting synergistically with the oxygen atoms of rhodamine, thereby increasing the sensitivity of the new two-dimensional sensor for detecting Ca^2+^. The sensing mechanism is based on a ratiometric signal readout. Upon binding with Ca^2+^, the emission peak of the MOF itself exhibits a blue shift and enhanced intensity, while the emission of the encapsulated Rhodamine B remains relatively stable. The ratio of these two emission intensities changes, which effectively eliminates environmental interference and enables highly accurate quantification. This specific recognition is attributed to the strong hard-hard acid-base interactions between Ca^2+^ and the basic azine groups within the confined pores of the MOF. In the presence of interfering cations at concentrations similar to plasma, the sensor exhibited extraordinary sensitivity and selectivity for Ca^2+^ detection [205].

Based on the aforementioned research, the general design strategies and core challenges of MOF-fluorescent dye composite sensors can be summarized. The core strategy involves constructing a host-guest architecture: the MOF serves as a rigid host, preventing dye molecule aggregation-caused quenching through pore confinement, and leveraging its functional sites (such as unsaturated metal sites and characteristic functional groups) for targeted recognition. Introducing dual emission centers to construct ratiometric sensors is a key strategy for enhancing anti-interference capability. The main current challenges lie in ensuring the stable immobilization of dyes within the MOF to prevent leakage, and precisely regulating the energy transfer pathways between components to achieve efficient ratiometric responses. These principles provide important guidance for developing new high-performance sensors.

#### 4.2.6. MOF-Other Nanomaterials

MOFs can also be combined with other nanomaterials, such as carbon nanotubes, graphene, and polymer nanoparticles, to further enhance the performance of sensors [206,207,208,209,210,211,212,213].

**Figure 7:**
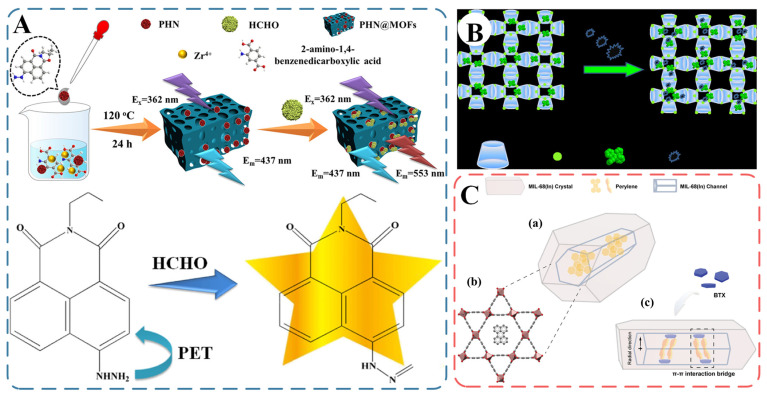
Examples of MOF-other nanoparticle composites for fluorescence sensing: (**A**) Schematic Illustration of the PHN@MOF Probe for the Detection of formaldehyde [214]. (**B**) Illustration of the TPE@γ-CD-MOF-K^+^ synthetic process [215]. (**C**) (**a**,**b**) LG@MOF Crystal Structure of Perylene@MIL-68(In) and Its Initial Molecular Configuration under Confinement of One-Dimensional Channels and (**c**) Proposed Sensing Mechanism of Perylene@MIL-68(In) Subject to BTX Molecules [216].

Wang et al. synthesized N-propyl-4-hydrazino-naphthalimide (PHN) as a fluorescent probe and embedded it into UiO-66-NH_2_ to obtain a nanocomposite material (PHN@MOF) (Figure 7A). The hydrazino groups of PHN act as reactive sites for formaldehyde, and through a condensation reaction, the reaction occurs rapidly, producing stable methylene hydrazine products. The sensor was used to construct a home device for formaldehyde monitoring. Additionally, the inherent fluorescence emission of UiO-66-NH_2_ provides a reference signal for formaldehyde detection. The detection operates on a fluorescence “turn-on” mechanism, where the embedded PHN probe reacts specifically with formaldehyde via a condensation reaction, inhibiting a photoinduced electron transfer process and restoring its fluorescence. This sensitivity is dramatically enhanced by the UiO-66-NH_2_ metal–organic framework, which employs a space confinement effect to selectively preconcentrate formaldehyde near the probe while excluding larger interfering molecules. The intrinsic blue fluorescence of the MOF itself serves as a stable reference, enabling ratiometric and visual detection through a distinct color change from blue to yellow under UV light [214].

Qiu et al. encapsulated the AIE material tetraphenylethene (TPE) in γ-cyclodextrin-MOF-K (γ-CD-MOF-K^+^) (Figure 7B). The γ-CD-MOF is an environmentally friendly material composed of naturally recyclable γ-CD and alkaline metal ions (such as K^+^). The prepared γ-CD-MOF-K^+^ has varying pore sizes, allowing it to simultaneously accommodate both TPE and explosives. Due to the MOF’s limiting effect on the vibrations of TPE, the photoluminescence (PL) of the TPE@γ-CD-MOF composite material is higher than that of TPE molecules alone. TPE@γ-CD-MOF-K^+^ exhibits sensitive fluorescence quenching activity towards nitroaromatic explosives in both liquid and solid states. The electron-rich TPE molecules inside the MOF transfer electrons to electron-deficient nitroaromatic explosives, which causes a decrease in fluorescence intensity. The unique multi-pore structure of the MOF allows both the TPE and the explosive molecules to be hosted simultaneously, enabling effective detection in both liquid and solid phases [215].

Zhang et al. synthesized a luminescent composite, perylene@MIL-68(In), by encapsulating perylene molecules into the metal-organic framework MIL-68(In) via a facile one-pot method (Figure 7C). In its solid state, the composite exhibits characteristic emission from the E-type excimer state of perylene. Upon exposure to BTX vapors (benzene, toluene, xylene), a distinct transformation from the E-excimer to the Y-excimer state occurs, induced by enhanced guest-host confinement and π-π interactions. This transformation results in a significant fluorescence enhancement (Turn-On response), enabling the use of this composite as a highly selective fluorescent sensor for BTX detection [216].

In order to better compare the performance of the fluorescence sensors based on MOF listed in Section 4, the following summary is presented in Table 3.

## 5. Conclusions and Future Perspectives

This review systematically summarizes recent advancements in MOF-based fluorescent sensors for rapid detection, covering their construction strategies, sensing mechanisms, and diverse applications. Owing to their high specific surface area, tunable pore structures, and readily functionalizable surfaces, MOF materials have emerged as an ideal matrix for constructing high-performance fluorescent sensing platforms. Through the rational selection of metal centers and organic ligands, or their strategic hybridization with functional units such as metal nanomaterials, COFs, and QDs, the sensitivity, selectivity, and stability of these sensors can be effectively tuned. This enables the efficient detection of various targets, including metal ions, biological molecules, pesticide residues, and environmental pollutants. Despite significant achievements, the transition from laboratory proof-of-concept to practical application and widespread commercialization faces several challenges and opportunities. Future research efforts should focus on the following interconnected strategic directions.

### 5.1. Material Innovation: Stability, Intelligence, and Integration

The long-term stability of MOF sensors in practical, complex environments—such as aqueous solutions, extreme pH, and biological fluids—remains a critical bottleneck. The core of future material innovation lies in moving beyond single-property optimization towards the synergistic enhancement of multiple attributes. Firstly, the intrinsic stability of MOFs under chemical, moisture, and photochemical conditions must be systematically improved, while also addressing cost concerns associated with expensive metals like lanthanides. This can be achieved by constructing robust coordination bonds based on the HSAB principle, introducing hydrophobic functional groups or protective layers, and implementing precise defect control. Secondly, a promising frontier involves transforming defects from “detrimental factors” into “design tools.” Controlled defect engineering can create more unsaturated metal sites to enhance recognition and modulate energy transfer pathways between ligands and metal centers, paving the way for a new generation of highly sensitive ratiometric fluorescent sensors [217]. Furthermore, developing MOFs with stimuli-responsive “smart pores” (triggered by pH, light, or target molecules) enables selective capture and release of analytes, offering breakthrough detection selectivity. The ultimate goal is to tailor specialized MOF materials that combine high stability, sensitivity, selectivity, and low cost for specific application scenarios, such as in vivo diagnostics and continuous environmental water monitoring.

### 5.2. Technology Fusion and Miniaturization: Towards Point-of-Care and High-Throughput Analysis

The capabilities of a single sensing material are ultimately limited. The deep integration of MOF sensors with cutting-edge biotechnology and micro/nano-fabrication technologies is an inevitable trend for building next-generation advanced detection systems. Microfluidic chips provide an ideal platform for integrating MOF sensors, enabling the automation and miniaturization of sample pretreatment, reaction, separation, and detection [218]. Immobilizing MOFs within microchannels allows for the construction of lab-on-a-chip systems that drastically reduce reagent consumption, enable parallel analysis of multiple targets, and significantly enhance portability. This is crucial for on-site rapid testing and applications in resource-limited settings. More frontier is the combination with CRISPR/Cas systems. MOFs can serve as efficient delivery vehicles for CRISPR components or as nanozymes for signal amplification, while the CRISPR system provides unparalleled nucleic acid recognition specificity. Pioneering research has demonstrated the feasibility of regulating MOF-based nanozyme activity via CRISPR-Cas12a for sensitive nucleic acid detection. This “MOF transduction + CRISPR recognition” strategy opens a new paradigm for detecting genetic biomarkers and pathogens [56,219,220]. Further integrating such systems with wearable devices—developing flexible MOF sensors embedded in textiles or patches—can facilitate the continuous, non-invasive monitoring of biomarkers in sweat or tears. Coupled with smartphones for data readout and analysis, this ultimately leads to intelligent sensing networks integrating materials, devices, and data clouds.

### 5.3. Application Expansion and Deepening: From Single-Target Detection to Complex System Analysis

With advancements in materials and platforms, the application scope of MOF sensors urgently needs to shift from relatively simple model systems to more challenging real-world scenarios. A core challenge is developing sensors capable of highly selective detection of low-abundance disease biomarkers (e.g., microRNA, specific proteins, metabolites) directly in complex biological matrices like whole blood, serum, or urine. This requires MOF surfaces with excellent anti-biofouling properties and the ability to effectively exclude interferents with similar structures through pore size sieving or tailored surface chemistry. On the other hand, single-analyte detection can no longer meet the demand for holistic assessment of complex systems, such as food spoilage, environmental pollutant classification, or disease subtyping. The future focus should be on developing MOF-based sensor arrays (“electronic tongues/noses”) [221,222]. By utilizing a series of MOFs with distinct chemical properties to generate unique fluorescent response “fingerprints,” and combining them with artificial intelligence (AI) and machine learning (ML) algorithms for pattern recognition of multi-dimensional data, the overall classification and identification of complex samples becomes possible (e.g., distinguishing different bacterial strains, assessing food safety levels) [223]. This represents a leap from mere “detection” to insightful “analysis”.

### 5.4. The Data-Driven Intelligent Future: AI-Powered Sensing Paradigms

The ultimate advancement of sensing technology is inseparable from the intelligence of data processing. MOF sensors, particularly ratiometric, multi-emissive, and array-based ones, generate massive and complex data streams. Traditional analytical methods struggle to fully exploit this potential. The introduction of AI and ML is poised to revolutionize this landscape. By training deep learning models, AI can accurately quantify analyte concentrations from complex fluorescence changes, effectively distinguish overlapping signals, and even predict outcomes in the presence of interferents, significantly enhancing analytical accuracy and reliability. Furthermore, AI can play a role in inverse design, assisting in predicting and designing new-generation MOF sensing materials with desired properties by analyzing the relationships between known MOF structures and sensing performance [224]. This fosters a closed-loop research paradigm: “material design → sensing application → data analysis → optimized design.”

In conclusion, as a vibrant interdisciplinary research field, MOF-based fluorescent sensors are progressively moving from proof-of-principle towards practical application. The path forward lies in the deep integration of material design, technology fusion, and intelligent analysis. Through interdisciplinary collaborative innovation aimed at systematically addressing the core challenges of stability, selectivity, and integration, it is reasonable to believe that MOF-based fluorescent sensors will play an increasingly critical role in clinical diagnostics, environmental monitoring, food safety, and national security in the near future. They offer powerful technological support for building a healthier and safer world.

## Figures and Tables

**Table 1:** Common metal centers, organic ligands and representative examples of several representative MOF series.

MOF Series	Common Metal Centers	Common Organic Ligands	Representative Examples
IRMOF	Zn^2+^, Cd^2+^	BDC and derivatives	IRMOF-1 (Zn_4_O-BDC), IRMOF-3 (BDC-NH_2_), IRMOF-16
ZIF	Zn^2+^, Co^2+^	Imidazolate ligands (e.g., 2-methylimidazole, 2-carboxyimidazole)	ZIF-8 (Zn-2-methylimidazole), ZIF-67 (Co-2-methylimidazole), ZIF-90
MIL	Fe^3+^, Cr^3+^, Al^3+^, V^3+^	Polycarboxylates (BDC, BTC, etc.)	MIL-53 (Al-BDC), MIL-88 (Fe-BDC), MIL-100 (Fe-BTC), MIL-101 (Cr-BDC)
PCN	Zr^4+^, Hf^4+^, Fe^3+^	Large π-conjugated polycarboxylates (e.g., TCPP, TCPB)	PCN-222 (Zr-TCPP), PCN-224, PCN-600
UiO	Zr^4+^, Hf^4+^	BDC and derivatives (NH_2_-BDC, NO_2_-BDC, etc.)	UiO-66 (Zr-BDC), UiO-67 (Zr-BPDC), UiO-68
HKUST	Cu^2+^	BTC	HKUST-1 (Cu-BTC)

**Table 2:** Examples of the fluorescence sensors based on different types of MOF composite materials and their detection mechanisms.

Types of MOF	Composite Materials	Detection Object	Detection Mechanism	Reference
Individual MOFs	PCN-224	Acrylamide	Fluorescence enhancement	[108]
	MnMOF	Glyphosate	Fluorescence quenching	[126]
	MOF-0.02TEA	Hydroquinone, Catechol	Fluorescence quenching	[111]
	In-MOF	Periodate	Fluorescence enhancement	[127]
	PCN-128	Oxytetracycline	Multiple fluorescence mechanisms coupling	[128]
	Eu-MOF	Trimethoprim		
	Tb-MOF	Ciprofloxacin		
	UiO-66-NH_2_	Zearalenone	Fluorescence quenching	[129]
	Ce-MOF	4-Nitrophenol	Fluorescence quenching	[130]
MOF-Metal Nanomaterials	Ag^+^/Eu^3+^@UiO-66(COOH)_2_	Tetracycline	Fluorescence quenching	[131]
	MOF-808-Al	Aminoglycosides	Multiple fluorescence mechanisms coupling	[132]
	Fe_3_O_4_@EuMOF	Fluoroquinolones	Fluorescence enhancement	[133]
MOF-on-MOF	Al-MOF@Eu-MOF@MIP	Aflatoxin B_1_	Multiple fluorescence mechanisms coupling	[134]
	Zn-BTC/IRMOF-3	Tetracyclines	Multiple fluorescence mechanisms coupling	[135]
MOF-COF	UIO-66-NH_2_@COF-B(OH)_2_	Transferrin	Fluorescence quenching	[136]
	NH_2_-MIL-53(Al)@TpTt-COF	Tetracycline	Multiple fluorescence mechanisms coupling	[137]
	UIO-66-NH_2_@TFPB-TAPA COF	Glutathione	Fluorescence quenching	[138]
	UiO-66-NH_2_@COF-BA	Methicillin-Resistant Staphylococcus aureus, Acinetobacter baumannii	Fluorescence enhancement	[58]
MOF-QDs	g-CNQDs@Zn-MOF	Riboflavin	Fluorescence quenching	[139]
	CQDs@Co/Mn-MOF	Norfloxacin	Fluorescence quenching	[140]
	Eu/CDs@MOF-808	Adenosine Triphosphate, Uric Acid	Multiple fluorescence mechanisms coupling	[141]
	CDs@ZIF-8(In)	Triethylamine	Fluorescence enhancement	[142]
MOF-Dye	Nile Blue@UIO-66-DHTA	Tobramycin	Multiple fluorescence mechanisms coupling	[143]
	RhB@PCN-222	Tetracyclines	Multiple fluorescence mechanisms coupling	[144]
	EY@MOF-5	Daclatasvir, Nitenpyram	Multiple fluorescence mechanisms coupling	[145]
MOF-Other Nanomaterials	Eu-MOF/carbon fiber	Al^3+^, H^+^	Fluorescence enhancement	[146]
	Zn-MOF/HOF	Doxycycline analogs	Multiple fluorescence mechanisms coupling	[147]
	Eu-BCA@SiO_2_-MIP	Zearalenone	Fluorescence quenching	[148]
	Ce-MOF/MWCNT	6-mercaptopurine	Fluorescence quenching	[149]
	CDs@Cu-MOF/TMTA-COF	Glyphosate	Multiple fluorescence mechanisms coupling	[150]
	Fe_3_O_4_@RhB@ZIF90@AChE	Organophosphorus, Carbamate Pesticides	Fluorescence enhancement	[151]

**Table 3:** Example of performance comparison of fluorescence sensors based on MOF.

Types of MOF	Composite Materials	Detection Object	Limit of Detection	Linear Range	Real Samples	Recovery Rate	Ref.
Individual MOFs	Cu@MOF	Alkaline Phosphatase	0.14 U/L	0.5–60 U/L	Human serum	101.19–107.90%	[153]
	Zr-MOF	Cr(VI)	1.4 µM	5–100 µM	Tap water	98.10–100.90%	[154]
	U-1 MOF	Perfluorooctanoic acid	1.68 μM (in suspension) 3.1 nM (on paper)	0–20 μM (suspension) 10–50 nM (paper)	Drinking water, Tap water	>91%	[155]
	Eu-MOF	Bromate (BrO_3_^−^)	3.7 × 10^−6^ mol/L	0–0.2 mM	Wheat flour	95.30–104.38%	[156]
MOF-Metal Nanomaterials	Au/MOF	Aflatoxin B_1_	Fluorescence mode: 0.07 ng mL^−1^ Visual mode: 0.08 ng mL^−1^	0.1–30 ng/mL	Rice, Corn, Wheat	Fluorescence mode: 97.9–111.5% Visual mode: 96.2–109%	[165]
	Fe/Eu-MOF	Alkaline Phosphatase	Colorimetric: 0.6 U/L Fluorescence: 0.9 U/L	1–200 U/L	Bovine, Chicken, Human serum	96.41–107.01%	[166]
	ZIF-8@CuNCs	Fenitrothion	Colorimetry: 2.00 μg/mL Fluorescence: 1.67 μg/mL	Colorimetry: 6–200 μg/mL Fluorescence: 5–40 μg/mL	Lake water, Tap water	Colorimetry: 97.90–104.17% Fluorescence: 98.17–101.35%	[167]
MOF-on-MOF	Tb^3+^@UIO66/MOF801	Fluoride ion	4.029 μM	0–0.6 mM	Tap water	/	[172]
	Zn/Fe-MOF@PDANSs (Zn-TCPP-MOF, MIL-101(Fe), PDANSS)	Sulfamethazine	0.025 ng/mL	0.5–100 ng/mL	Chicken, Pork	92.69–108.48%	[157]
	Fe_3_O_4_@SiO_2_@MOF-on-MOF	SARS-CoV-2 N protein	0.13 pg/mL	10^−12^–10^−6^ g/mL (1 pg/mL–1 µg/mL)	Simulated saliva	98.79–102.53%	[173]
MOF-COF	AMC@ZIF-90/3D-COF	Chloroquine Phosphate	7.4 × 10^−6^ M	4 × 10^−5^–4 × 10^−4^ M	Urine	/	[180]
			5.2 × 10^−7^ M	3 × 10^−6^–4 × 10^−5^ M	Serum	/	
		Folic Acid	3.5 × 10^−6^ M	1 × 10^−5^–1 × 10^−3^ M	Milk	/	
	MOF@COF–B(OH)_2_@MIP	Transferrin	57.6 nM (Fluorescence) 2.7 nM (Electrochemical)	0.1–40 μM (Fluorescence) 0.05–30 μM (Electrochemical)	Human serum, Human urine	84.3–103.9%	[181]
	UiO-66-NH_2_@COF	Fluoride ion	/	1–500 μM	/	/	[136]
MOF-QDs	SQDs@MOF-5-NH_2_	Patulin	0.753 pg/mL	1 pg/mL–100 ng/mL	Apple juice	89.03–107.67%	[74]
	BNCDs/Tb-MOF@GR5 DNAzyme	Pb^2+^	0.96 ppb (2.89 nM)	2–1000 nM	Water	92.76–119%	[62]
	N-CDs@Eu-MOF@MIP (BR@MIP)	Malathion	0.05 μM	1–10 μM	Lettuce, Tap water, Soil	93.0–99.3%	[193]
MOF-Dye	RhB@ZIF-8	Nitrofurantoin	0.012 µM	10^−6^–10^−10^ M	Rainwater, Lake water, Tap water	88.45–129.25%	[202]
		Oxytetracycline	8.9 nM				
	BODIPY@Eu-MOF	Fluoride ion	0.1737 µM	0–30 µM	/	/	[203]
		Hydrogen peroxide	6.22 nM	0–6 µM	/	/	
		Glucose	6.92 nM	0–6 µM	Serum	96.5–103.2%	
	Zr-AzoMOF	Dufulin	2.96 ng/mL	0–5 µg/mL	Paddy water, Paddy soil, Cucumber, Polished rice	90.35–109.84%	[204]
	TMU-5S	Calcium ion	17 nM	1 × 10^−6^ M–5 × 10^−5^ M	Water	/	[205]
MOF-Other Nanomaterials	TPE@γ-CD-MOF-K	Nitroaromatics	~3 ppm	0–30 ppm	/	/	[215]
	PHN@MOF	Formaldehyde	0.173 µM	1–4 µM	Indoor Air, Liquor	93.33–105.00%	[214]
	Perylene@MIL-68(In)	Xylene	1300 ppm	0–8000 ppm	Simulated Contaminated Air	/	[216]

## Data Availability

No primary research results, software, or code have been included, and no fresh data were generated or analyzed as part of this review.

## Abbreviations

The following abbreviations are used in this manuscript:

MOFs	Metal-organic frameworks
IRMOFs	Isoreticular Metal-organic Frameworks
ZIFs	Zeolitic Imidazolate Frameworks
MILs	Materials of Institut Lavoisier
COFs	Covalent organic frameworks
QDs	Quantum dots
PCNs	Porous Coordination Networks
UiO	University of Oslo
CPLs	Coordination Pillared-Layer
BDC	Benzene-1,4-dicarboxylic acid
HPLC	High-performance liquid chromatography
GC	Gas chromatography
MS	Mass spectrometry
ALP	Alkaline phosphatase
ATP	Adenosine triphosphate
PPD	p-Phenylenediamine
IFE	Inner filter effect
PDI	Pentamethylene diisocyanate
PFAS	Per- and polyfluoroalkyl substances
PFOA	Perfluorooctanoic acid
AFB_1_	Aflatoxin B_1_
TMB	Tetramethylbenzidine
cDNA	Complementary DNA
PPi	Pyrophosphate
Pi	Phosphate
NCs	Nanocages
FRET	Fluorescence resonance energy transfer
N protein	Nucleocapsid protein
MNCs	Metal nanocluster
Au NCs	Gold nanoclusters
AgAu NCs	Gold-silver alloy nanoclusters
PDANSs	Polydopamine
SMZ	Sulfamethoxazole
AMC	7-amino-4-methylcoumarin
CQP	Chloroquine phosphate
FA	Folic acid
scCHA	Self-circulating catalytic hairpin assembly
SQDs	Sulfur quantum dots
PAT	Patulin
PVP	Polyvinyl pyrrolidone
APTES	(3-Aminopropyl)triethoxysilane
CDs	Carbon dots
BNCDs	Boron-nitrogen-doped carbon dots
Mal	Malathion
RhB	Rhodamine B
NFT	Nitrofurantoin
OTC	Oxytetracycline
FA	Formaldehyde
ACP	Acidic phosphatase
TPE	Tetraphenylethene
OPD	O-phenylenediamine
DOX	Doxycycline
Arg	L-arginine
PEI	Polyethyleneimine
DAP	2,3-diaminophenazine
AAP	Ascorbic acid 2-phosphate
MIP	Molecularly imprinted polymer
TEA	Triethylamine
BTC	1,3,5-Benzene tricarboxylic acid
TCPP	5,10,15,20-Tetrakis(4-carboxyphenyl)porphyrin
TCPB	1,2,4,5-Tetrakis(4-carboxyphenyl)benzene
MMM	Mixed-membrane matrix
PET	Photoinduced electron transfer
AGs	Aminoglycoside antibiotics
ACQ	Aggregation-caused quenching
